# Carbon Nanotube (CNT) Honeycomb Cell Area-Dependent Optical Reflectance

**DOI:** 10.3390/nano6110202

**Published:** 2016-11-07

**Authors:** Junthorn Udorn, Akimitsu Hatta, Hiroshi Furuta

**Affiliations:** 1 Department of Electronic and Photonic Systems Engineering, Kochi University of Technology, Tosayamada-cho, Kami, Kochi 782-8502, Japan; 178002a@gs.kochi-tech.ac.jp (J.U.); hatta.akimitsu@kochi-tech.ac.jp (A.H.); 2 Center for Nanotechnology, Research Institute, Kochi University of Technology, Tosayamada-cho, Kami, Kochi 782-8502, Japan

**Keywords:** carbon nanotube (CNT), CNT honeycomb structures, total, diffuse, specular reflectances

## Abstract

The relationship between the physical structure of carbon nanotube (CNT) honeycomb structures and their total, diffuse, and specular reflectance is investigated for the first time. It is found that CNT honeycomb structures with average cell areas of smaller than 30 μm^2^ show a higher total reflectance. Particularly, a thinner, highly packed CNT (buckypaper) film, along with a larger wall height and higher ratio of wall height to cell area, markedly increase the total reflectance for cell areas smaller than 30 μm^2^, which means that a higher total area of buckypapers in CNT walls and bottom areas increases the total reflectance, including the diffuse reflectance. It is also found that the total reflection of non-absorbed light in CNT honeycomb structures consists primarily of diffuse reflectance.

## 1. Introduction

The unique morphologies and structures of carbon nanotubes (CNTs) have received much attention for optical and electronic applications because CNTs have extraordinary photonic properties, high electrical current endurability, and mechanical stiffness [1,2,3,4]. In addition, the morphologies of CNT forests can be modified to enhance charge generation, separation, and transport in optical-electronic applications [5]. For modifying CNT forest morphologies, liquid or vapor treatment is a simple, economic method that provides a high yield [5,6]. The liquid treatment of CNTs exhibits self-assembly, where one-dimensional material forms into three-dimensional micro or macro structures with various morphologies [5]. Previous papers have reported that the liquid and vapor treatment onto multi-walled CNTs (MWCNTs) exhibits the self-assembly of hierarchical networks to form honeycomb structures due to capillary forces arising during solution evaporation [7,8,9,10]. The larger surface area of such honeycomb structures is expected to allow the efficient assembly of sensitized nanoparticles of quantum dots (QDs), which can serve as an electrode scaffold to capture and transport photo-generated electrons in solar cells [11]. Additionally, the spacing in the structure of silicon solar cells with CNT honeycomb top electrodes allows higher transmission of light to photo-active parts of solar cells when light irradiation is perpendicular to the substrate [12]. Moreover, wall-shaped condensed CNT films can serve as an “electron-carrying highway”, enhancing high conductivity to an electrode of solar cells [5].

The total reflectance of randomly oriented CNT-compressed sheets is more than 80% for a CNT film thicknesses of 0.3–1 μm [13], while for high nanotube forests (300–500 μm in height) it is 1%–2% across a range from UV to mid IR (200–2000 nm) [14]. Yang et al. reported an extraordinarily low total reflectance of 0.045% for a mat of vertically aligned multi-walled carbon nanotubes (VA-MWCNTs) forests in a visible region at wavelengths of 457–633 nm [15]. Theoretically, the reflectance of CNT forests can be explained by the fact that, for light incidence on a forest top surface of CNTs of small angle with respect to the CNT axis, electrons on the CNT body cannot couple with the electric fields, which provides a weak optical interaction between the CNT forests and normally incident light resulting in a lower total reflectance [16].

For photovoltaic applications, the optical properties of the materials are one of the most important parameters for achieving light enhancement. Recently, the reuse of the optical reflectance of existing light to significantly increase the efficiency in dye sensitized solar cells (DSSC) has been reported [17]. It is expected that solar cells using CNTs can be designed so that the highly reflected light from the CNTs is absorbed by sensitizers, generating a larger number of electron-hole pairs. To date, no applications making use of the optical reflectance of CNT honeycomb structures have been reported. In this paper, the relationship between the physical structure of CNT honeycomb structures and the total, diffuse, and specular reflectance of the CNT honeycomb structures is presented for the first time. CNT honeycomb structures were fabricated and the cell areas were controlled by a simple method of ethanol treatment. The total, diffuse, and specular reflectance of CNT honeycomb structures was then studied. In particular, the physical structure including cell area, wall height to whole area ratio (surface area), bottom area to whole area ratio (total bottom area with respect to whole cell area), wall height, and buckypaper film thickness was investigated to show the influence of the parameters of the physical structure on the reflectance.

## 2. Materials and Methods

CNT forests were prepared using a method from our previous work, with a CNT density of ~8.0 × 10^9^/cm^2^ and wall heights of ~10 μm by a 20-s synthesis on AlO*_x_*/Fe bi-layered catalyst films (30 nm/1 nm in thickness) on a silicon substrate using a thermal chemical vapor deposition (thermal-CVD) system at 730 °C with a C_2_H_2_ gas source at 54 Pa [18]. In order to fabricate honeycomb structures and control the cell areas, CNT forest samples with of 1.5 cm^2^ area were treated with varying volumes of 98.5% ethanol from 5, 10, 15, 20, 25, and 30 μL in an ambient environment until the ethanol was completely evaporated. The longer evaporation times of larger volumes of ethanol provided larger average cell areas of CNT honeycomb structures (see Supplementary Figure S1). The morphologies of as-synthesized CNT forests and CNT honeycomb structures were characterized by field-emission scanning electron microscope (FE-SEM) (JEOL JSM-5310, JEOL Ltd., Tokyo, Japan). Evaluation of the cell areas, wall heights, bottom areas, and buckypaper film thicknesses at the bottom areas was quantitatively performed by the image processing software ImageJ (National Institutes of Health (NIH), Bethesda, MA, USA) [19]. The total and diffuse reflectance from UV through to visible regions (190–900 nm) was measured using a spectrophotometer (HITACHI U-3900, HITACHI high-technologies, Tokyo, Japan) with an integrating sphere.

## 3. Results and Discussion

### 3.1. Morphologies of CNT Honeycomb Structures

**Figure 1:**
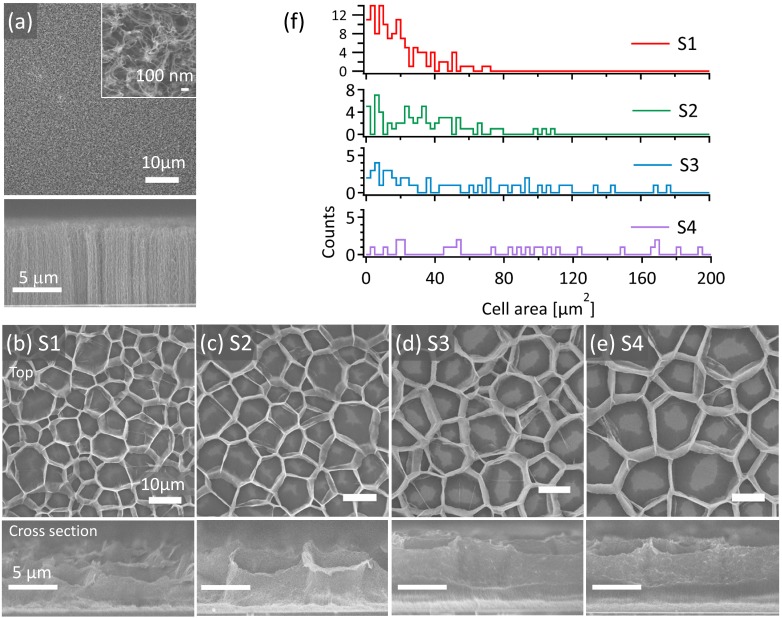
(**a**) Top-view field-emission scanning electron microscope (FE-SEM) micrographs of as-synthesized carbon nanotubes (CNTs). A highly magnified image of vertically aligned CNT forests of as-synthesized CNT forests is displayed in the upper-right inset and a cross-section is displayed in the lower image; Top-view (above) and cross-section (below) of FE-SEM micrographs of CNT honeycomb structures for samples (**b**) S1 (average cell area 16 μm^2^); (**c**) S2 (34 μm^2^); (**d**) S3 (51 μm^2^); (**e**) S4 (97 μm^2^); (**f**) Histogram of CNT honeycomb cell area.

Figure 1a displays a top-view of an FE-SEM micrograph of as-synthesized CNTs with mean diameters of 10–15 nm. The lower image of Figure 1a shows a cross section displaying vertically aligned CNTs with heights of ~10 μm. The CNT forests show a uniformly flat surface (Figure 1a), in which the CNTs exhibit random entangles with each other, tip bending, and a loosely connected random surface, as shown in the larger magnification in the inset of Figure 1a. FE-SEM images and cell area histograms for honeycomb CNT samples S1, S2, S3, and S4 with average cell areas of 19, 34, 51, and 97 μm^2^ are shown in Figure 1b–f, respectively. After ethanol treatment, self-assembling honeycomb structures were formed by attractive forces of aggregation due to capillary forces [7,8,9,10], as can be seen in the top-view of the CNT forests in Figure 1b–e. A honeycomb cell comprises two main parts, vertically standing CNT walls and a collapsed CNT sheet on the bottom area. These CNT walls and the collapsed CNT mat on the bottom area are composed of so-called CNT buckypapers.

As shown in the lower image of Figure 1b, honeycomb cells of sample S1 are polygons, including a few triangle patterns and mainly quadrangular, pentagon, and hexagon structures, with wall heights varying from 2.9 to 4.4 μm (3.7 μm in average). The average cell area of the CNT honeycomb structures for S1 is 19 μm^2^ and the cell area distribution is shown in the red histogram of Figure 1f. As depicted for each honeycomb cell for all S1–S4 samples, the bottom area at the center shows a catalyst composed silicon substrate, which is especially clear in Figure 1d,e. The thicknesses of the CNT buckypaper films near the silicon area at the center toward the CNT wall are 0.47 μm in average in S1, as shown in the lower image of Figure 1b. Sample S2 shows a larger average cell area of 34 μm^2^ with a higher average wall height of 3.7 μm and average CNT buckypaper film thickness of 0.58 μm, as displayed in the lower image of Figure 1c. Sample S3 shows a much larger average cell area of 51 μm^2^ with a higher average wall height of 7.2 μm and average CNT buckypaper film thickness of 0.89 μm, as displayed in Figure 1d. Sample S4 shows the largest honeycomb average cell area of 97 μm^2^ with the largest average wall heights of 7.9 μm, shown in the cross-sectional image of Figure 1e, and CNT buckypaper film thicknesses varying from 1.0 μm.

### 3.2. Total, Diffuse, and Specular Reflectance

**Figure 2:**
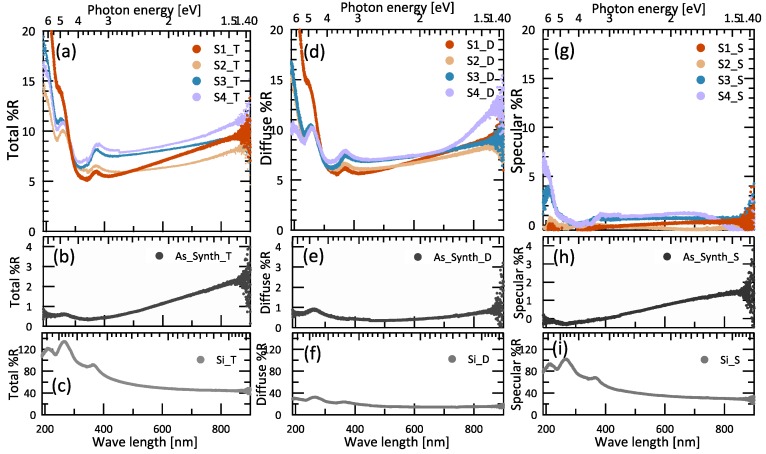
Total reflectance (Total %R), diffuse reflectance (Diffuse %R), and specular reflectance (Specular %R) of CNT honeycomb structures as a function of UV-visible wavelengths (nm) and photon energy (eV) for S1 (red), S2 (orange), S3 (blue), and S4 (violet) in panels (**a**,**d**,**g**); as-synthesized CNT forests (black) in panels (**b**,**e**,**h**); and a silicon substrate (grey) in panels (**c**,**f**,**i**), respectively.

When light is incident on a vertically aligned CNT forest, it can be absorbed, transmitted, and reflected by individual CNTs. The total reflectances of an as-synthesized CNT forest, four samples of CNT honeycomb structures (S1–S4), and the silicon substrate are plotted in Figure 2a–c. The as-synthesized vertically aligned CNT forest with a height of 10 μm exhibits a low average total reflectance of 0.5% (also see Table 1) in the UV region, as shown in Figure 2b. This result is similar to previous findings that show a low reflectance of 1%–2% across the UV region (190–380 nm) due to a multiplication of light reflectivity regardless of the very high nanotube forests of 300–500 μm [14]. The dip in the reflectance curve at a wavelength of 236 nm (5.3 eV) corresponds to an absorption peak attributed to a π-plasmon peak of CNTs [16]. As shown in Figure 2b, in the visible region (380–900 nm), the average total reflectance of the as-synthesized CNT forest is 1.0%, which is higher than that in the UV region. As Murakami et al. [16] reported, vertically-aligned CNTs have a lower absorption for lower photon energy in the visible region, which supports our result for as-synthesized CNTs showing that the total reflectance is higher in the visible region than in the UV region.

After ethanol treatment, the average total reflectance in the UV region for the CNT honeycomb structures in samples S1–S4 increased to 9.5%–11%, as compared with that for an untreated sample of 0.5%. Two silicon peaks were observed for CNT honeycomb structures at 270 and 380 nm, attributed to the silicon substrate [20,21]. These silicon peak heights were reduced by the light absorption of the CNT-formed honeycomb structures. In the visible region of 380–900 nm, the total reflectance of the CNT honeycomb structures in S1–S4 has lower average values of 7.1%–8.4% compared with the UV region. The total, diffuse, and specular reflectance in the UV region exhibit values higher than in the visible region, as shown in Figure 2a,d,g. This behavior is due to Rayleigh scattering, which provides a higher reflectance at shorter wavelengths [22]. As can be seen in Figure 2a, the smaller cell area of S1 gives a higher total reflectance than for the larger cell areas of S2, S3, and S4 in the UV region, but shows a somewhat lower total reflectance in the visible region, as shown in Figure 2a. This result is analyzed further using data from additional samples with reference to the physical parameters of the honeycomb structures in Section 3.3 and Section 3.4.

**Figure 3:**
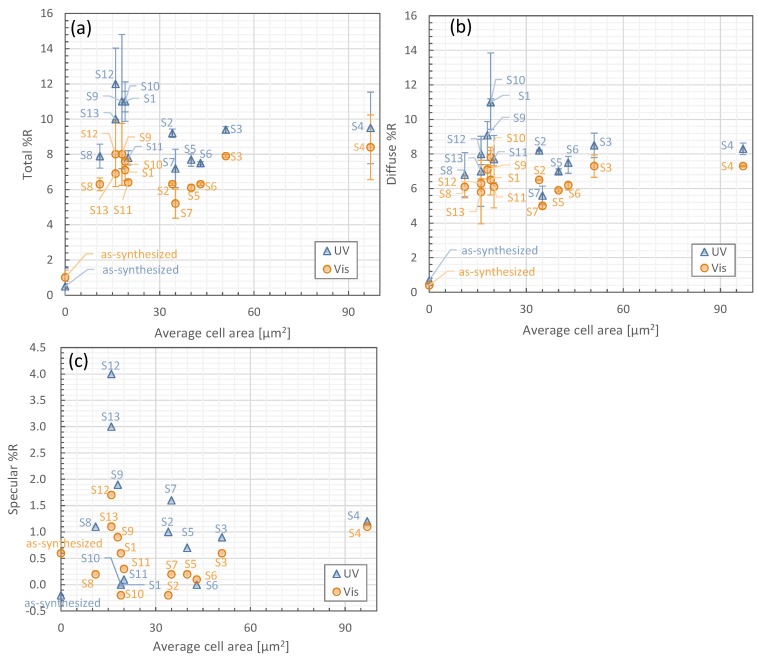
(**a**) Total reflectance (Total %R); (**b**) Diffuse reflectance (Diffuse %R); and (**c**) Specular reflectance (Specular %R) as a function of average honeycomb cell area.

The diffuse reflectances of as-synthesized CNT forests, four samples of CNT honeycomb structures (S1–S4), and the silicon substrate are shown in plots Figure 2d–f. The diffuse reflectance of as-synthesized CNTs shows lower values than those of the CNT honeycomb structures, of 0.7% in the UV region and 0.4% in the visible region. For the CNT honeycomb structures, the diffuse reflectance of S1 with the smallest cell area surprisingly shows a higher value of 11% than those of S2, S3, S4, and the as-synthesized CNT forests in the UV region, and exhibits the lowest reflectance of 6.5% in the visible region, as shown in Figure 3b. Interestingly, the diffuse reflectances of S1–S4 are closer to each other than is the case for the total reflectance. Again, these results are further analyzed in Section 3.3 and Section 3.4.

The specular reflectances, calculated as the difference between the total and diffuse reflectance, of as-synthesized CNT forests, S1–S4, and the silicon substrate are plotted in Figure 2g–i. The specular reflectance of as-synthesized CNTs shows a value of 0.2% in the UV region and 0.6% in the visible region. For CNT honeycomb structures, the specular reflectance of S1 is 0.0%, while the larger cell areas of S2, S3, and S4 give 0.0%, 0.9%, and 1.2%, respectively in the UV region. In the visible region, the specular reflectance of samples S1–S4 exhibit values up to 1.1%. In the CNT honeycomb structures due to the high diffuse reflectance, the specular reflectance is very small compared to the total and diffuse reflectances. Further analysis of the relationship between cell areas and the specular reflectance is given in Section 3.3 and Section 3.4.

### 3.3. CNT Honeycomb Cell Areas vs. Total, Diffuse, and Specular Reflectance

Figure 3a shows the average total reflectance in the UV and visible regions as a function of the averaged CNT honeycomb cell areas for samples S1–S13, which is also summarized in Table 1. Two groups of samples based on cell area can be recognized in Figure 3a. The first group, S2–S7, with cell areas larger than 30 μm^2^, shows a trend of increasing total reflectance for increasing cell area. For the second group, S1, S9, S10, S12, and S13, with cell areas smaller than 30 μm^2^, the total reflectance shows higher values of about 10%–12% in the UV region and about 7%–8% in the visible region. The diffuse and specular reflectance follows the same trend as observed for total reflectance in Figure 3b,c, respectively. The specular reflectance of cell areas smaller than 30 μm^2^ shows increased values, especially in S12 at 4.0% and 1.7% in the UV and visible regions, respectively. We investigate further the result showing higher total and diffuse reflectance for cell areas smaller than 30 μm^2^ by examining how the physical structure represented by the wall height to whole area ratio, bottom area to whole area ratio, wall height, and buckypaper film thickness influences the total and diffuse reflectance in Section 3.4.

Figure 3c shows very small values for the specular reflectance for S6, namely a very small difference of 0.1% in the visible region and 0% in the UV region, showing that the total and diffuse reflectances in the UV region are the same. As we noted in Section 3.2, our study shows that the difference between the total and diffuse reflectance is very small, especially so for S1. It can be interpreted that the honeycomb structure of cells creates a rough surface with regard to reflecting light, and thus the light is mainly scattered as diffuse reflectance.

### 3.4. High Reflectance of Cell Areas Smaller Than 30 μm^2^

**Figure 4:**
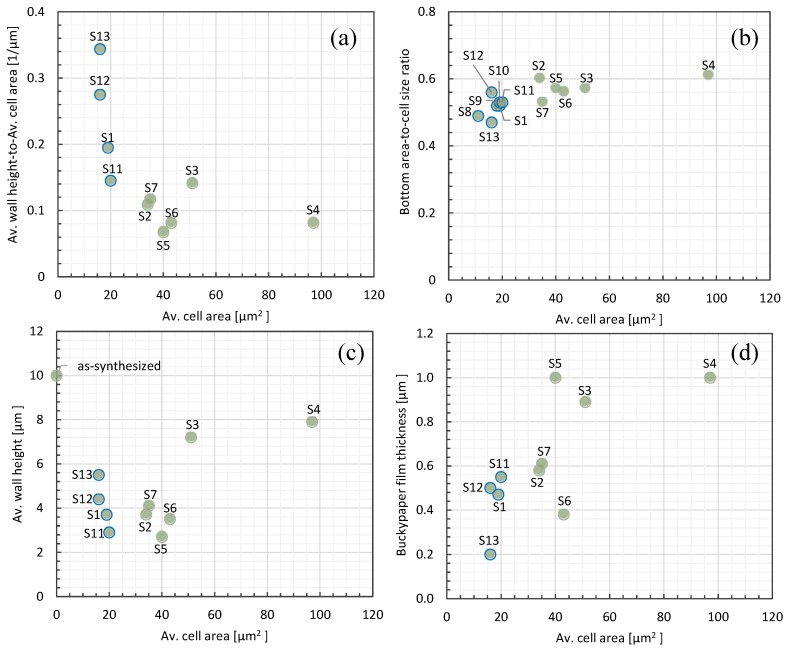
(**a**) Average wall height to cell area ratio; (**b**) Bottom area to whole area ratio; (**c**) Average wall height; and (**d**) Average buckypaper film thickness as a function of average cell area. Samples with average cell areas of less than 30 μm^2^ are indicated by green circles with blue outlines.

**Figure 5:**
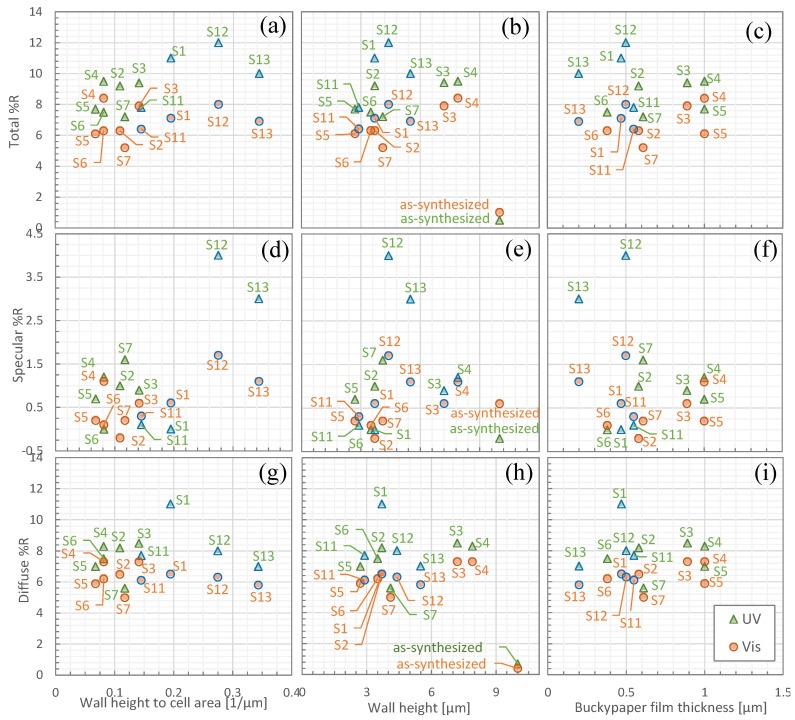
Total reflectance (Total %R) as a function of (**a**) Wall height to cell area; (**b**) Wall height; and (**c**) Buckypaper film thickness; Specular reflectance (Specular %R) as a function of (**d**) Wall height to cell area; (**e**) Wall height; and (**f**) Buckypaper film thickness; Diffuse reflectance as a function of (**g**) Wall height to cell area; (**h**) Wall height; and (**i**) Buckypaper film thickness. UV and visible wavelengths are indicated by green triangles and orange circles, respectively. Samples with a cell area of less than 30 μm^2^ are indicated with blue outlines.

In order to investigate the higher total, diffuse, and specular reflectance in cell areas smaller than 30 μm^2^, we consider the physical structure of the honeycomb networks. Figure 4a shows the wall height to cell area ratio (surface area) as a function of cell area. The ratio of wall height to cell area is higher in cell areas smaller than 30 μm^2^ (indicated by green circles with blue outlines), with S13 (average cell area 16 μm^2^) having the highest ratio of 0.34 μm^−1^. In particular, as displayed in Figure 5a, the height to whole area ratio in S1, S12, and S13 corresponds to a high total reflectance in both the UV (green triangles) and visible (orange circles) regions. Moreover, Figure 5d shows that S12 and S13 have high specular reflectance in both the UV and visible regions. Thus, the study shows that a higher wall height and a very small cell area (larger surface area) give a higher reflectivity, resulting in higher total, diffuse, and specular reflectances. However, other CNT honeycomb physical structures may also lead to a high total and specular reflectance, and they are investigated below.

This relationship between the total reflectance and the wall height to cell area ratio may help explain the high total reflectance of S1 compared to S2–S4 in the UV region. Meanwhile, in the visible region, S1 has the lower total reflectance than S2–S4 because of a lower ratio of wall height to cell area. As shown in Figure 5g, the diffuse reflectance is not dependent on wall height to cell area.

Figure 4b shows a plot of the bottom area to whole area ratio as a function of cell area. The average cell area of 16 μm^2^ in S13 with the high total reflectance provides a lower ratio of 0.47 μm. (See more in Supplementary Figure S2). A lower specular reflectance is expected for a lower ratio of bottom area to whole area for cell sizes smaller than 30 μm^2^ because the higher CNT walls with smaller bottom areas will interfere with the specular reflectance. However, our results show the opposite, with a higher specular and total reflectance for the group with smaller cell areas with a lower ratio of bottom area to whole area. To interpret this unexpected result, the wall height as a function of cell area is analyzed as follows. Based on the plot of the average wall height as a function of cell area in Figure 4c, S13, with a cell area smaller than 16 μm^2^ and a high total reflectance, exhibits the highest wall heights of 5.5 μm of samples with area less than 30 μm^2^. Accordingly, Figure 5b shows the wall heights of S1 (3.7 μm), S12 (4.4 μm), and S13 (5.5 μm) corresponding to high total reflectances of 11%, 12%, and 10%, respectively. From the trend shown in Figure 5b, the higher total reflectance of S1 compared with S2–S4 in the UV region cannot be due to a higher wall height. In contrast, in the visible region, the lower total reflectance of S1 than S2–S4 may be due to a lower wall height. Figure 5e shows that S12 and S13 have high specular reflectance. This can be explained in that the wall height of highly packed CNTs, formed in cells smaller than 30 μm^2^, can serve as glassy carbon of high reflectance, increasing the total and specular reflectance (see Supplementary Figure S3). Hence, it can be concluded that a higher wall height increases the specular and total reflectance for cell areas smaller than 30 μm^2^.

Figure 4d shows the buckypaper film thickness at the bottom area as a function of cell area. The cell area of 16 μm^2^ in S13 gives a thinner buckypaper film thicknesses of 0.20 μm, and the thinner buckypaper film thickness in S13 gives a high total reflectance of 10%, especially in the UV region. Meanwhile films with thicknesses greater than 0.5 μm show a total reflectance lower than 10% in the visible region, as shown in Figure 5c. This result shows a correspondence with Shabaneh et al.’s findings [13] that the total reflectance of CNT buckypaper films increases with decreased CNT film thicknesses. This behavior can be interpreted as indicating a high reflectance for high-density, thinner-thickness CNT films because the penetration depth of the evanescent field is expected to be small for high-density CNT films. Moreover, Figure 5f shows that S12 and S13 have high specular reflectance with thinner buckypaper films. Therefore, thinner buckypaper films can increase the total and specular reflectance. The high total reflectance of S1 compared with S2–S4 in the UV region can be explained as being due to thinner buckypaper films. The total reflectance of S1 is lower as compared with S2–S4 in the visible region is caused by thicker buckypaper films.

## 4. Conclusions

This study investigated the controlling of cell area in a CNT honeycomb structure by a simple method of ethanol treatment, in which the average cell area could be decreased by a shorter evaporation time of ethanol. The total, diffuse, and specular reflectance of CNT honeycomb structures was investigated. Cell areas smaller than 30 μm^2^ with a 3–8 μm wall height showed a higher total reflectance of 6%–12% in the UV region and 6%–8% in the visible region, where as-synthesized CNT forests exhibited corresponding values of 0.5% and 1.0% in the UV and visible regions, respectively. In particular, our findings highlighted that thinner buckypaper films of high-density CNTs in cooperation with a higher ratio of wall height to cell area (larger surface area) and wall height increases the total and specular reflectance. In addition, we found that the highest measured diffuse reflectance of 11% in the UV region, as well as the total reflectance, is likely strongly influenced by a higher total area of buckypapers in CNT walls and bottom areas. Interestingly, this study found that the main component of total reflectance from CNT honeycomb structures is diffuse reflectance. In future work, it is expected that a higher total reflectance will be obtained for larger surface areas, which will contribute to the achievement of efficient absorption of light into quantum dots to improve the efficiency of QD solar cells utilizing CNT electrodes.

## Figures and Tables

**Table 1:** Comparison of parameters of as-synthesized CNTs and samples S1–S13 of CNT honeycomb structures. R_T_: total optical reflectance, R_D_: diffuse optical reflectance, UV: ultraviolet, Vis: visible.

Samples	% R (UV)		% R (Vis)		Cell Size (μm^2^)	Wall Height to Cell Size Ratio	Bottom Area to Cell Size Ratio	Wall Height (μm)	Buckypaper Film Thickness (μm)
	R_T_	R_D_	R_T_	R_D_					
as-synthesized CNTs	0.5	0.7	1.0	0.4	-	-	-	~10	-
S1	11	11	7.1	6.5	19	0.20	0.52	3.7	0.47
S2	8.2	8.2	6.3	6.5	34	0.11	0.60	3.7	0.58
S3	9.4	8.5	7.9	7.3	51	0.14	0.57	7.2	0.89
S4	9.5	8.3	8.4	7.3	97	0.08	0.61	7.9	1.0
S5	7.7	7.0	6.1	5.9	40	0.07	0.57	2.7	1.0
S6	7.5	7.5	6.3	6.2	43	0.08	0.56	3.5	0.38
S7	6.2	5.6	5.2	5.0	35	0.12	0.53	4.1	0.61
S8	7.9	6.8	6.3	6.1	11	-	0.49	-	-
S9	11	9.1	8.0	7.1	18	-	0.52	-	-
S10	11	11	7.6	7.8	19	-	0.53	-	-
S11	7.8	7.7	6.4	6.1	20	0.14	0.53	2.9	0.55
S12	12	8	8	6.3	16	0.28	0.56	4.4	0.5
S13	10	7	6.9	5.8	16	0.34	0.47	5.5	0.2

## Supplementary Materials

The following are available online at http://www.mdpi.com/2079-4991/6/11/202/s1, Figure S1: Average cell areas vs. ethanol evaporation time, Figure S2: Total, diffuse, specular reflectance vs. honeycomb physical structures, Figure S3: FE-SEM of CNT honeycomb walls.

## References

[1] Iijima S. (1991). Helical microtubules of graphitic carbon. Nature.

[2] Avouris P., Freitag M., Perebeinos V. (2008). Carbon-nanotube photonics and optoelectronics. Nat. Photonics.

[3] Tang Z.K., Zhang L., Wang N., Zhang X.X., Wen G.H., Li G.D., Wang J.N., Chan C.T., Sheng P. (2001). Superconductivity in 4 Angstrom Single-Walled Carbon Nanotubes. Science.

[4] Walters D.A., Ericson L.M., Casavant M.J., Liu J., Colbert D.T., Smith K.A., Smalley R.E. (1999). Elastic strain of freely suspended single-wall carbon nanotube ropes. Appl. Phys. Lett..

[5] Cui K., Chiba T., Omiya S., Thurakitseree T., Zhao P., Fujii S., Kataura H., Einarsson E., Chiashi S., Maruyama S. (2013). Self-assembled microhoneycomb network of single-walled carbon nanotubes for solar cells. J. Phys. Chem. Lett..

[6] Kenan S., Ahang Y., Meng J., Green E.C., Tajaddod H., Li H., Minus M.L. (2013). Structural polymer-based carbon nanotube composite fibers: understanding the processing-structure-performance relationship. Materials.

[7] Lim X., Gary Foo H.W., Chia G.H., Sow C.H. (2010). Capillarity-assisted assembly of carbon nanotube microstructures with organized initiations. ACS Nano.

[8] Chakrapani N., Wei B., Carrillo A., Ajayan P.M., Kane R.S. (2004). Capillarity-driven assembly of two-dimensional cellular carbon nanotube foams. Proc. Natl. Acad. Sci. USA.

[9] De Volder M., Tawfick S.H., Park S.J., Copic D., Zhao Z., Lu W., Hart A.J. (2010). Diverse 3D microarchitectures made by capillary forming of carbon nanotubes. Adv. Mater..

[10] De Volder M., Hart A.J. (2013). Engineering Hierarchical Nanostructures by Elastocapillary Self-Assembly. Angew. Chem. Int. Ed..

[11] Chen J., Li C., Zhao D.W., Lei W., Zhang Y., Cole M.T., Chu D.P., Wang B.P., Cui Y.P., Sun X.W. (2010). A quantum dot sensitized solar cell based on vertically aligned carbon nanotube templated ZnO arrays. Electrochem. Commun..

[12] Li C., Xia J., Wang Q., Chen J., Li C., Lei W., Zhang X. (2013). Photovoltaic property of a vertically aligned carbon nanotube hexagonal network assembled with CdS quantum dots. ACS Appl. Mater. Interfaces.

[13] Shabaneh A.A., Girei S.H., Arasu P.T., Rashid S.A., Yunusa Z., Mahdi M.A., Paiman S., Ahmad M.Z., Yaacob M.H. (2014). Reflectance Response of Optical Fiber Coated With Carbon Nanotubes for Aqueous Ethanol Sensing. IEEE Photonics J..

[14] Mizuno K., Ishii J., Kishida H., Hayamizu Y., Yasuda S., Futaba D.N., Yumura M., Hata K. (2009). A black body absorber from vertically aligned single-walled carbon nanotubes. Proc. Natl. Acad. Sci. USA.

[15] Yang Z.-P., Ci L., Bur J.A., Lin S.-Y., Ajayan P.M. (2008). Experimental observation of an extremely dark material made by a low-density nanotube array. Nano Lett..

[16] Murakami Y., Einarsson E., Edamura T., Maruyama S. (2005). Polarization dependence of the optical absorption of single-walled carbon nanotubes. Phys. Rev. Lett..

[17] Lee J.Y., Lee S., Park J.-K., Jun Y., Lee Y.-G., Kim K.M., Yun J.H., Cho K.Y. (2010). Simple approach for enhancement of light harvesting efficiency of dye-sensitized solar cells by polymeric mirror. Opt. Express.

[18] Koji H., Furuta H., Sekiya K., Nitta N., Harigai T., Hatta A. (2013). Increased CNT growth density with an additional thin Ni layer on the Fe/Al catalyst film. Diam. Relat. Mater..

[19] Schneider C.A., Rasband W.S., Eliceiri K.W. (2012). NIH Image to ImageJ: 25 Years of image analysis. Nat. Methods.

[20] Green M.A., Keevers M.J. (1995). Optical properties of intrinsic silicon at 300 K. Prog. Photovolt. Res. Appl..

[21] Green M.A. (2008). Self-consistent optical parameters of intrinsic silicon at 300 K including temperature coefficients. Sol. Energy Mater. Sol. Cells.

[22] Yu Z., Brus L. (2001). Rayleigh and Raman Scattering from Individual Carbon Nanotube Bundles. J. Phys. Chem. B.

