# Supplementary material for: Carbon Nanotube (CNT) Honeycomb Cell Area-Dependent Optical Reflectance
Source: Nanomaterials (Basel). 2016 Nov 7;6(11):202. doi: 10.3390/nano6110202 (PMC5245753; doi:10.3390/nano6110202)
Supplement: Supplementary file 1 [file nanomaterials-06-00202-s001.docx]

Supplementary Materials: Carbon Nanotube (CNT) Honeycomb Cell Area-Dependent Optical Reflectance

Junthorn Udorn, Akimitsu Hatta and Hiroshi Furuta


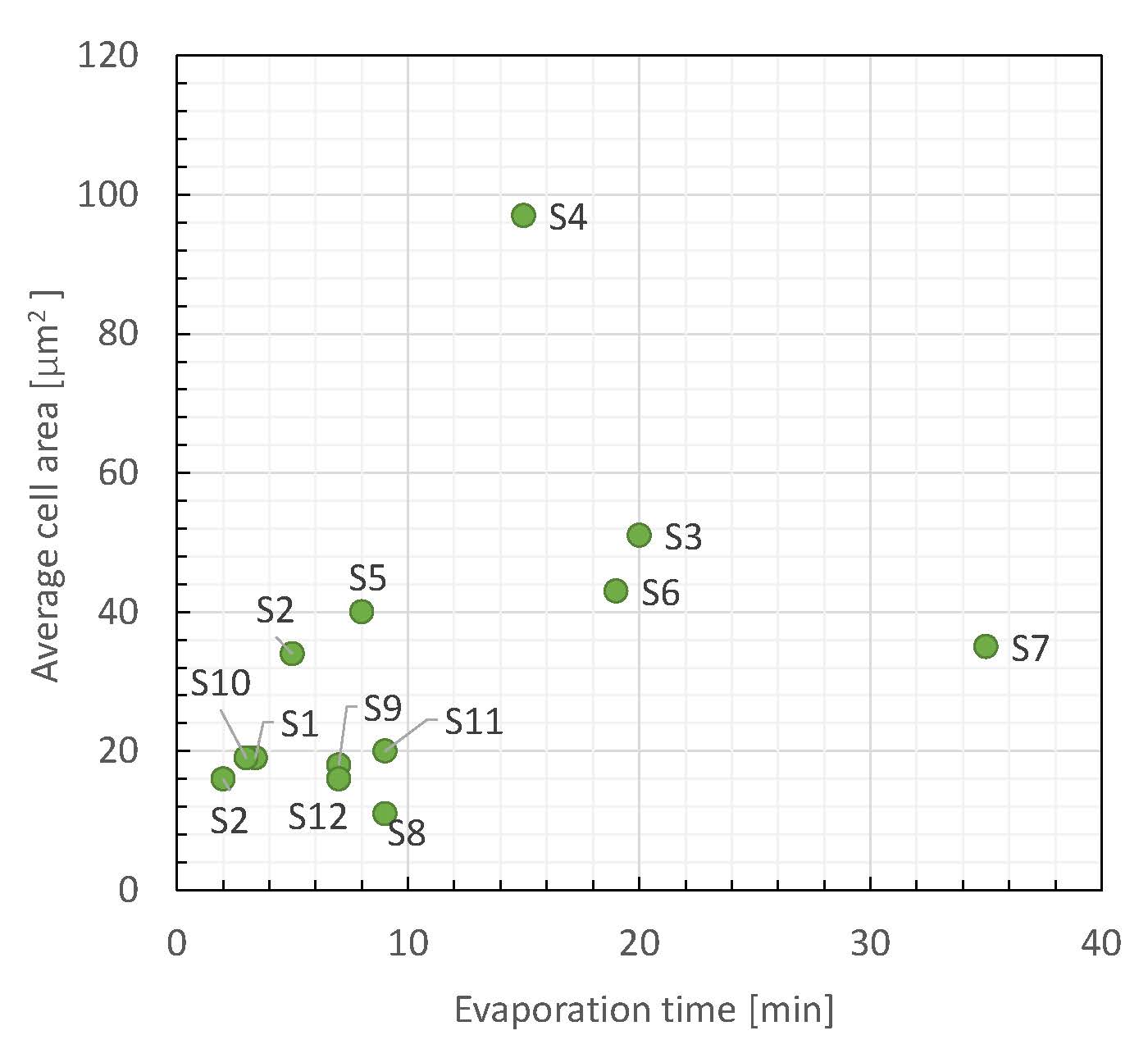


**Figure S1.** Average cell areas vs. ethanol evaporation time. A plot of average cell areas as a function of ethanol evaporation time at the room temperature during fabrication of carbon nanotube (CNT) honeycomb structures.

**Figure S2.** Total, diffuse, specular reflectances vs. CNT honeycomb physical structures. Plots of total (**a**–**d**), diffuse (**e**–**h**) and specular (**i**–**l**) reflectance vs. physical properties of CNT honeycomb: wall height to cell area ratio, bottom to cell size ratio, wall height, and buckypaper film thickness.


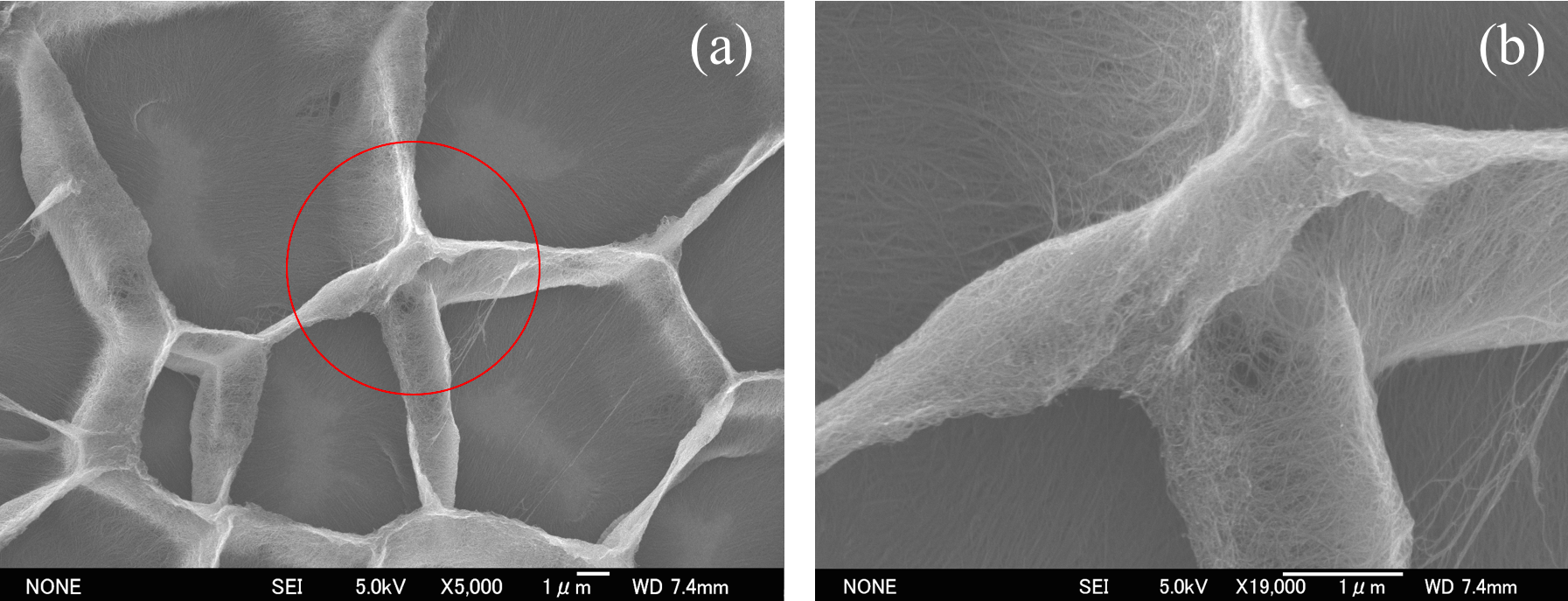


**Figure S3.** Field-emission scanning electron microscope (FE-SEM) of CNT honeycomb walls. FE-SEM images of (**a**) CNT honeycomb walls and (**b**) magnified CNT honeycomb walls.

© 2016 by the authors. Submitted for possible open access publication under the
terms and conditions of the Creative Commons Attribution (CC-BY) license (http://creativecommons.org/licenses/by/4.0/).
